# Assessing *Trans*-Inhibition of OATP1B1 and OATP1B3 by Calcineurin and/or PPIase Inhibitors and Global Identification of OATP1B1/3-Associated Proteins

**DOI:** 10.3390/pharmaceutics16010063

**Published:** 2023-12-31

**Authors:** John T. Powell, Ruhul Kayesh, Alexandra Ballesteros-Perez, Khondoker Alam, Pascaline Niyonshuti, Erik J. Soderblom, Kai Ding, Chao Xu, Wei Yue

**Affiliations:** 1 Department of Pharmaceutical Sciences, University of Oklahoma Health Sciences Center, Oklahoma City, OK 73117, USA; john-powell@ouhsc.edu (J.T.P.);; 2 Proteomics and Metabolomics Core Facility, Duke University School of Medicine, Durham, NC 27708, USA; 3 Department of Biostatistics & Epidemiology, University of Oklahoma Health Sciences Center, Oklahoma City, OK 73104, USA; kai-ding@ouhsc.edu (K.D.); chao-xu@ouhsc.edu (C.X.)

**Keywords:** drug transport, drug–drug interactions, trans-inhibition, organic anion transporting polypeptide, OATP1B1, OATP1B3, calcineurin, cyclosporine A, tacrolimus, SCY-635

## Abstract

Organic anion transporting polypeptide (OATP) 1B1 and OATP1B3 are key determinants of drug–drug interactions (DDIs). Various drugs including the calcineurin inhibitor (CNI) cyclosporine A (CsA) exert preincubation-induced *trans*-inhibitory effects upon OATP1B1 and/or OATP1B3 (abbreviated as OATP1B1/3) by unknown mechanism(s). OATP1B1/3 are phosphoproteins; calcineurin, which dephosphorylates and regulates numerous phosphoproteins, has not previously been investigated in the context of preincubation-induced *trans*-inhibition of OATP1B1/3. Herein, we compare the *trans*-inhibitory effects exerted on OATP1B1 and OATP1B3 by CsA, the non-analogous CNI tacrolimus, and the non-CNI CsA analogue SCY-635 in transporter-overexpressing human embryonic kidney (HEK) 293 stable cell lines. Preincubation (10–60 min) with tacrolimus (1–10 µM) rapidly and significantly reduces OATP1B1- and OATP1B3-mediated transport up to 0.18 ± 0.03- and 0.20 ± 0.02-fold compared to the control, respectively. Both CsA and SCY-635 can *trans*-inhibit OATP1B1, with the inhibitory effects progressively increasing over a 60 min preincubation time. At each equivalent preincubation time, CsA has greater *trans*-inhibitory effects toward OATP1B1 than SCY-635. Preincubation with SCY-635 for 60 min yielded IC_50_ of 2.2 ± 1.4 µM against OATP1B1, which is ~18 fold greater than that of CsA (0.12 ± 0.04 µM). Furthermore, a proteomics-based screening for protein interactors was used to examine possible proteins and processes contributing to OATP1B1/3 regulation and preincubation-induced inhibition by CNIs and other drugs. A total of 861 and 357 proteins were identified as specifically associated with OATP1B1 and OATP1B3, respectively, including various protein kinases, ubiquitin-related enzymes, the tacrolimus (FK506)-binding proteins FKBP5 and FKBP8, and several known regulatory targets of calcineurin. The current study reports several novel findings that expand our understanding of impaired OATP1B1/3 function; these include preincubation-induced *trans*-inhibition of OATP1B1/3 by the CNI tacrolimus, greater preincubation-induced inhibition by CsA compared to its non-CNI analogue SCY-635, and association of OATP1B1/3 with various proteins relevant to established and candidate OATP1B1/3 regulatory processes.

## 1. Introduction

Organic anion transporting polypeptides (OATPs) 1B1 and OATP1B3 are expressed on the sinusoidal membrane of hepatocytes in the liver and mediate the hepatic uptake of numerous therapeutic drugs and endogenous compounds [1]. The concurrent administration of drugs that are potent OATP inhibitors, such as rifampicin [2] and cyclosporine A (CsA) [3,4], has been reported to increase systemic exposure of OATP substrates such as statins and is associated with statin-induced myopathy [5,6].

Preincubation-induced *trans*-inhibitory effects [7,8,9,10,11] on OATP1B1 and/or OATP1B3 (abbreviated as OATP1B1/3) have been reported in vitro by many drugs/compounds, where OATP1B1/3-mediated substrate transport is reduced after inhibitor preincubation and washing, when determined in the absence of the inhibitor in the transport assay. OATP1B1 and OATP1B3 are phosphorylated proteins [7,12]. Some kinase modulator drugs/compounds such as protein kinase C activator [7] and tyrosine kinase inhibitor nilotinib [13,14] down-regulate OATP1B1/3 transport function under *trans*-inhibition conditions via modulating kinase activity. Many of the inhibitors with *trans*-inhibitory effects on OATP1B1/3 were also reported to cause time-dependent inhibition of OATP1B1/3 [3,4,8,9,10,11,15,16], where inhibitor preincubation enhances the inhibitory effect of the inhibitor, resulting in a reduced IC_50_/inhibition constant (K_i_). Among inhibitors with *trans*- and/or time-dependent inhibitory effects on OATP1B1/3, the immunosuppressant calcineurin inhibitor (CNI) CsA has garnered the most attention, being highlighted in the US FDA final guidance to industry [17], due to its importance in causing clinically significant DDIs against many OATP1B1/3 drug substrates including statins [5,18,19,20,21,22,23,24,25,26,27]. 

**Figure 1:**
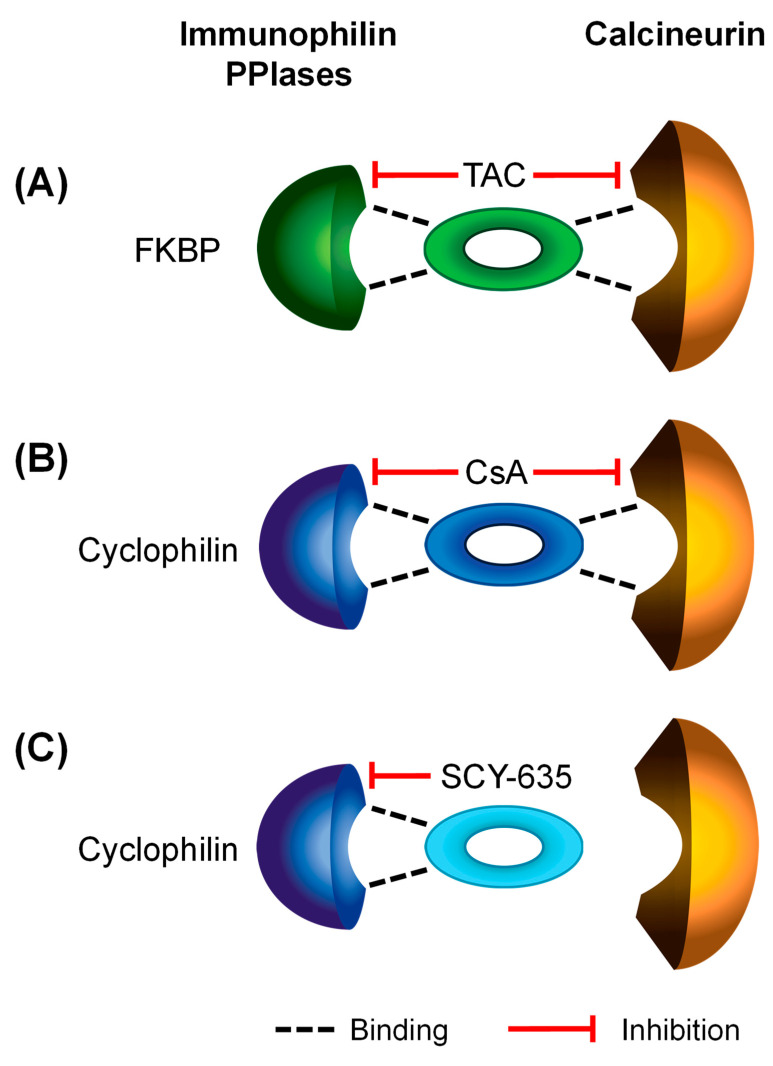
Schematic representation of the binding and inhibition of immunophilins and/or calcineurin by tacrolimus (TAC) (**A**), CsA (**B**), and the non-immunosuppressive cyclophilin inhibitor SCY-635 (**C**).

Two CNIs, CsA and tacrolimus (also called FK506), have been used in immunosuppression therapy [28]. Calcineurin is a protein phosphatase that regulates the activities of various protein targets via dephosphorylation [29,30]. CsA- and tacrolimus-mediated immunosuppression is achieved by blocking the calcineurin-mediated dephosphorylation of nuclear factor of activated T cells (NFAT), resulting in the subsequent activation of NFAT [31]. Meanwhile, CsA and tacrolimus are peptidyl-prolyl cis/trans isomerase (PPIase) inhibitors [32,33,34]. The cellular receptors (also known as immunophilins) of respective CNIs, cyclophilins for CsA and FK506-binding proteins (FKBPs) for tacrolimus, are PPIases [35,36]. PPIases catalyze the conversion of the peptide bond proceeding an internal proline residue from *cis* to *trans*, thereby accelerating slow, rate-limiting steps in the folding of many proteins [33,37]. As depicted in Figure 1A,B (adapted from review by Schiene-Fischer [38]), CsA and tacrolimus bind to cyclophilins (e.g., cyclophilin A [33]) or FKBPs (e.g., FKBP12 [34]), respectively, and form binary complexes that inhibit the immunophilins’ PPIase activities. Such a binary complex is a prerequisite for the formation of the ternary complex between the CNI (CsA or tacrolimus), its respective immunophilin receptor (a cyclophilin or an FKBP), and calcineurin, whereby the phosphatase activity of calcineurin is inhibited. 

Due to the important role of the cyclophilins’ PPIase activity in various biological processes including protein folding, post-translational modification, and cell signaling, cyclophilins regulate numerous protein activities [39,40,41,42,43,44,45,46,47,48]. They are implicated in several diseases including viral infections, with human immunodeficiency virus (HIV)-1 and hepatitis C virus (HCV) being notable examples [38]. Considerable attention has been given to develop non-immunosuppressive cyclophilin inhibitors—i.e., chemical entities that preserve the potent PPIase inhibitory activity while lacking calcineurin inhibition activity (as depicted in Figure 1C), for the treatment of HCV and other diseases [38]. The most advanced compounds of this type are two CsA derivatives, alisporivir [49,50] and SCY-635 [32,51], which have both been shown to reduce viral RNA levels within the plasma of HCV-infected patients during treatment in clinical trials. Although competitive inhibition of OATP1B1 and OATP1B3 by alisporivir has been reported [52], preincubation-induced *trans*-inhibitory effects of a non-immunosuppressive cyclophilin inhibitor on OATP-mediated transport has not been reported to date. 

Currently, tacrolimus is the preferred CNI over CsA in most transplant recipients, largely due to the risk of DDIs of CsA against statins [53,54]. Notably, false-negative prediction of OATP-mediated DDIs has been reported for tacrolimus [55]. Although tacrolimus does not cause DDIs against atorvastatin [20] or simvastatin [27], DDIs of tacrolimus have been reported against the OATP1B1 substrates [56,57] cerivastatin and pravastatin, in which long-term treatment of tacrolimus increased the area under the curve of the plasma concentration time profile (AUC) of cerivastatin by 1.5-fold in renal transplant recipients [58] and that of pravastatin by ~10-fold in two pediatric and adolescent recipients who received cardiac transplants [25]. In addition, rhabdomyolysis was noted in a case report of a liver transplant recipient receiving rosuvastatin and tacrolimus [59]. A prior study reported no preincubation-induced *trans*-inhibition of tacrolimus against OATP1B1 and OATP1B3 [4]. However, this study used estrone-3-sulfate (E_1_S) as the substrate for OATP1B1; E_1_S has been reported to be less sensitive in assessing OATP1B1 inhibition [60]. Therefore, it is necessary to reevaluate the *trans*-inhibitory effects of the CNI tacrolimus on OATP1B including using an OATP1B1 substrate (e.g. E217βG) proven to be more sensitive in assessing OATP1B1 inhibition [60]. Also, assessing the *trans*-inhibitory effects of the CsA-analogous non-immunosuppressive cyclophilin inhibitor SCY-635 in comparison with CsA may yield valuable information regarding potential mechanism(s) involved in CsA *trans*-inhibition on OATP1B1. 

Protein–protein interactions (PPIs) play important roles in numerous biological processes, with most protein biological functions being achieved through PPIs [61]. Currently, only limited information exists in this regard. OATP1B1 was recently reported to be associated with the adapter protein PSD-95/Discs Large/ZO-1 domain containing 1 (PDZK1), and such interaction is required for OATP1B1 trafficking to the plasma membrane [62]. OATP1B3 can form hetero-oligomers with OATP1B1, Na^+^/taurocholate co-transporting polypeptide (NTCP) [63], and organic cation transporter (OCT1) [64]. To date, global identification of OATP1B1- and OATP1B3-associated proteins through mass spectrometry-based proteomics has not been reported. As many drugs with reported *trans*-inhibitory effects on OATP1B1/3 are kinase inhibitors [10,11,13,14,65], the identification of proteins associated with OATP1B1 and OATP1B3 will not only broaden our understanding to transporter function and regulation in general, it may also shed light on potential mechanism(s) underlying the *trans*-inhibition of OATP1B1/3. 

The aims of the current study are two-fold: (1) to determine the preincubation-induced *trans*-inhibitory effects of tacrolimus and SCY-635, a CsA derivative non-immunosuppressive cyclophilin inhibitor, on OATP1B1/3-mediated transport in comparison with the effects of CsA; and (2) to discover OATP1B1- and OATP1B3-associated-proteins using immunoprecipitation-coupled mass spectrometry (IP-MS), with an emphasis on identifying proteins that are linked to reported OATP1B1/3 regulation mechanisms [66] and preincubation-induced *trans*-inhibition of OATP1B1/3.

## 2. Materials and Methods

### 2.1. Materials

[^3^H]-cholecystokinin 8 (CCK-8, specific activity 88.0 Ci/mmol), [^3^H]-estradiol 17 β-D-glucuronide (E_2_17βG, specific activity 41.4 Ci/mmol), and [^3^H]-estrone-3-sulfate (E_1_S, specific activity 54.0 Ci/mmol) were purchased from Perkin Elmer Life Science (Waltham, MA, USA). Unlabeled pitavastatin and [^3^H]-pitavastatin (specific activity 5 Ci/mmol) were purchased from American Radiolabeled Chemicals (St. Louis, MO, USA). Unlabeled substrates (CCK-8, E_2_17βG, and E_1_S), ionomycin calcium salt, dimethyl sulfoxide (DMSO), Hanks’ Balanced Salt Solution (HBSS), Dulbecco’s Modified Eagle Medium (DMEM), antibiotic antimycotic solution, trypsin-EDTA solution, and Triton X-100, cOmplete™ protease inhibitor cocktail and PhosSTOP™ phosphatase inhibitor were purchased from Sigma-Aldrich (St. Louis, MO, USA). Fetal bovine serum (FBS) was purchased from Hyclone Laboratories (Logan, UT, USA). Poly-L-lysine was purchased from Trevigen, Inc. (Gaithersburg, MD, USA). Geneticin was purchased from Life Technologies (Grand Island, NY, USA). Bio-Safe II liquid scintillation mixture was purchased from Research Products International (Mt. Prospect, IL, USA). All other materials were purchased from Thermo Fisher Scientific (Waltham, MA, USA). The SCY-635 (CAS No. 210759-10-7) was purchased from Waterstone Pharmaceuticals (HuBei) Co., LTD (Tianmen, China). In addition to the certificate of analysis from the manufacturer, SCY-635 was authenticated by verifying its identity and purity using high-resolution (400 MHz) nuclear magnetic resonance (NMR) at the core facility of the University of Oklahoma. 

### 2.2. Cell Culture and Transfection

HEK293 stable cell lines overexpressing FLAG-tagged OATP1B1 (HEK293-FLAG-OATP1B1) [67], FLAG-tagged OATP1B3 (HEK293-FLAG-OATP1B3) [68], GFP-Myc-FLAG-OATP1B1 (HEK293-GFP-OATP1B1) [11], and GFP-Myc-FLAG-OATP1B3 (HEK293-GFP-OATP1B3) [11] as well as the HEK293-mock [69] cell lines were published previously. These stable cell lines were maintained in complete DMEM supplemented with 10% FBS (*v/v*), 1% antibiotic antimycotic solution, and 600 µg/mL Geneticin in a humidified atmosphere (95% O_2_, 5% CO_2_) at 37 °C. HeLa cells were cultured in DMEM containing 10% FBS.

### 2.3. Human Sandwich-Cultured Hepatocytes (SCH)

Fresh primary human hepatocytes were purchased from Lonza Walkersville, Inc. (Walkersville, MD, USA). Donor information is provided in Supplementary Table S1. Cell viability was 96% after centrifugation. Culture of human SCH was performed as described previously [7,67]. In brief, on day 1, cells were plated at 3 × 10^5^ cells per well in 24-well Biocoat™ plates (Thermo Fisher Scientific, Waltham, MA, USA). When cells had attached to the plate after 6 h of seeding, cells were overlaid with Matrigel™ (Thermo Fisher Scientific, Waltham, MA, USA) (0.25 mg/mL) in feeding medium: phenol red-free DMEM supplemented with 2 mM L-glutamine, 1% (*v/v*) MEM NEAA, 100 units/mL of penicillin G sodium, 100 g/mL of streptomycin sulfate, 0.1 μM dexamethasone, and 1% (*v/v*) ITS^+^ premix. Uptake experiments were conducted on day two of culture, the next day after seeding.

### 2.4. Purification of FLAG-OATP1B1 and FLAG-OATP1B3 by Immunoprecipitation and Immunoblot

HEK293- FLAG-OATP1B1, -FLAG-OATP1B3, and -Mock cells were seeded in poly-L-lysine coated dishes at a seeding density of 6.5 × 10^6^ cells per dish (145 mm diameter), 10 dishes each, and were allowed to grow for 48 h at 37 °C to confluence. Cells were washed once with cold PBS (12 mL per dish) and lysed with 2 mL ice-cold lysis buffer containing 50 mM Tris-HCl (pH 7.4), 150 mM NaCl, 1 mM EDTA, and 1% Triton X-100, which was further supplemented with cOmplete^TM^ protease inhibitor cocktail and PhosSTOP^TM^ phosphatase inhibitor. Lysates were centrifuged for 10 min at 15,000 rpm at 4 °C, and supernatants were collected. M2 resin (270 µL) was washed twice with 1 mL TBS buffer at 4 °C, added to each of the lysate supernatants, and incubated with the supernatants overnight at 4 °C. After incubation, resin was washed seven times with lysis buffer (1 mL each time), vigorously shaking and then centrifuging at 8000 rpm for 1 min for each wash. After the last wash, the resin was incubated with 150 µL of the 2× sample buffer (125 mM Tri-HCl, pH 6.8, 4% SDS) at 60 °C for 10 min. After centrifuging at 8000 rpm for 30 s, supernatants were collected and stored at −80 °C. To verify the quality of the purification, 23 µL and 10 µL (1:10 diluted) eluted supernatant were loaded to each well of a 4–12% NuPAGE Bis-Tris protein gels for Colloidal blue staining per the manufacturer’s instructions and immunoblot with anti-FLAG antibody, respectively. The eluted samples were subjected to subsequent proteomics analysis.

### 2.5. Time-Lapse Confocal Fluorescence Microscopy

Effects of CsA (1.2 µM) on plasma membrane localization of GFP-OATP1B1 and GFP-OATP1B3 were qualitatively determined by time-lapse confocal live-cell imaging in HEK293-GFP-OATP1B1 and -1B3 cells, respectively, similarly as previously published [11] using an Olympus UPLSAPO 60× water immersion objective (N/A 1.2) with 473 nm laser excitation and an emission bandpass of 490–590 nm. Images were captured every 20 min from −19 min to 61 min. 

### 2.6. Qualitative Proteomics

Samples in 2× sample buffer (125 mM Tri-HCl, pH 6.8, 4% SDS) were spiked with 1 pmol bovine casein as an internal digestion control, reduced with 10 mM dithiothreitol for 20 min at 55 °C, alkylated with 25 mM iodoacetamide for 45 min at room temperature, and subjected to S-trap (Protifi, Fairport, NY, USA) trypsin digestion using manufacturer recommended protocols. Digested peptides were lyophilized to dryness and resuspended in 12 µL of 0.2% formic acid/2% acetonitrile containing 25 fmol/µL pre-digested yeast alcohol dehydrogenase (ADH)—MassPREP ADH Digestion Standard (Waters Corp., Milford, MA, USA)—as a technical control. Each sample was subjected to chromatographic separation on a Waters Mclass UPLC equipped with a 1.8 μm ACQUITY high strength silica (HSS) T3 C18 75 μm × 250 mm column (Waters Corp., Milford, MA, USA) with a 90 min linear gradient of 5–30% acetonitrile with 0.1% formic acid at a flow rate of 400 nanoliters/minute (nL/min) with a column temperature of 55 °C. Data collection on a Thermo Fusion Lumos mass spectrometer equipped with a FAIMS Pro ion-mobility device was performed for three different compensation voltages (−40 v, −60 v, −80 v). Within each compensation voltage (CV), a data-dependent acquisition (DDA) mode of acquisition with an r = 120,000 (@ *m/z* 200) full MS scan from m/z 375–1500 with a target automatic gain control (AGC) value of 1 × 10^4^ ions was performed. MS/MS scans with higher-energy C-trap dissociation (HCD) settings of 30% were acquired in the linear ion trap in “rapid” mode with a target AGC value of 1e4 and max fill time of 35 ms. The total cycle time for each CV was 0.66 s, with total cycle times of 2 s between like full MS scans. A 20 s dynamic exclusion was employed to increase depth of coverage. The total analysis cycle time for each sample injection was approximately 2 h.

Raw LC-MS/MS data files were processed in Proteome Discoverer 3.0 (Thermo Fisher Scientific, Waltham, MA, USA) and then submitted to independent Sequest database searches against a human protein database containing both forward (20,371 entries) and reverse entries of each protein. Search tolerances were 2 ppm for precursor ions and 0.8 Daltons for product ions using trypsin specificity with up to two missed cleavages. Carbamidomethylation (+57.0214 Da on cysteine) was set as a fixed modification, whereas oxidation (+15.9949 Da on methionine) was considered a dynamic mass modification. All searched spectra were imported into Scaffold (v5.3, Proteome Software, Portland, OR, USA), and scoring thresholds were set to achieve a maximum peptide false discovery rate of 1% using the PeptideProphet algorithm [70]. Data were output as total spectral matches. Raw data and corresponding Scaffold file have been uploaded to ProteomeXchange under the following submission: PXD044692 (username: reviewer_pxd044692@ebi.ac.uk, password Hk7DujFe).

### 2.7. Uptake Studies and IC_50_ Determination

HEK293-FLAG-OATP1B1 and HEK293-FLAG-OATP1B3 cells were seeded at a density of 1.5 × 10^5^ cells per well of 24-well plate coated with poly-L-lysine and allowed to grow for 48 h to confluence before uptake studies were performed as published previously; the preincubation effects on OATP1B1- and OATP1B3-mediated transport were also determined as previously published [7,11]. In brief, the cells were preincubated in complete culture medium with 10% FBS and 1% antibiotic antimycotic that contained either vehicle control (0.1% DMSO), tacrolimus (1 and 10 µM), SCY-635 (0.001–10 µM), or CsA (0.001–10 µM) for the designated times. After washing three times with HBSS buffer (pH 7.4), substrate accumulation was determined in the absence of inhibitors. [^3^H]-E_2_17βG (1 µM, 2 min) and [^3^H]-E_1_S (1 µM, 2 min) were used as probe substrates for OATP1B1; [^3^H]-CCK-8 (1 µM, 3 min) was used as the probe substrate for OATP1B3, similar to previous publications [7,11,67]. [^3^H]-pitavastatin accumulation (1 µM, 30 s) in human SCH was conducted on day 2 of culture as has been published previously [67]. Substrate accumulation was determined by scintillation counting (LS6500 scintillation counter, Beckman Coulter, Brea, CA) and normalized to a protein concentration determined via BCA assay (Pierce Chemical, Rockford, IL, USA). Transport assays on a blank plate without cells were conducted to correct for the nonspecific binding to the assay plate.

For IC_50_ determination, the substrate accumulation was expressed as percentage of the vehicle control was plotted against the SCY-635 or CsA preincubation concentrations (C). The IC_50_ values were estimated by nonlinear regression using the three-parameter model as below with GraphPad Prism v.9.0 (GraphPad Software, La Jolla, CA, USA).

*E* = *Bottom* + (*Top* − *Bottom*)/(*1* + (*C*/*IC*_50_))

### 2.8. Data Analysis

Statistical analyses were conducted using SAS software (version 9.4, Cary, NC, USA). As indicated in the figure legend, fold changes and associated standard errors (SEs) were estimated by linear mixed effect models or generalized linear mixed models using the log link function, a random effect (experiment date), and a fixed effect (treatment time or group), allowing for treatment time/group-specific variations [67]. When conducting multiple comparisons, the *p*-values were corrected using Bonferroni’s method. A two-sided *p*-value of <0.05 defines statistical significance. 

## 3. Results

### 3.1. Comparison of Preincubation Effects of SCY-635 and CsA on OATP1B1-Mediated Transport

**Figure 2:**
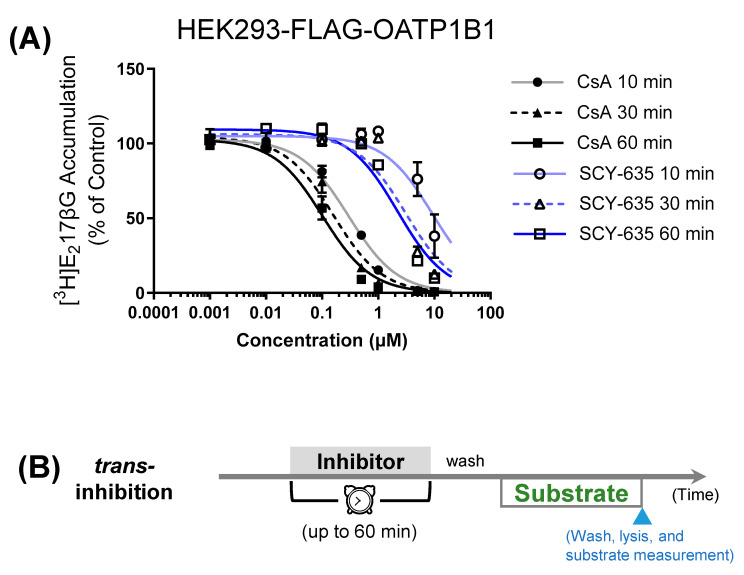
Effects of preincubation with CsA and SCY-635 on OATP1B1-mediated transport. (**A**) HEK293-FLAG-OATP1B1 cells were preincubated with vehicle control, CsA (0.001–10 µM), or SCY-635 (0.001–10 µM) for 10, 30, and 60 min. After washing, OATP1B1-mediated transport of [^3^H]-E_2_17βG was determined in the absence of inhibitor and expressed as percentage of vehicle control treatment. The IC_50_ values were determined by fitting dose–response curves to the data by nonlinear regression and are summarized in Table 1. Black solid (CsA, 60 min), black dashed (CsA, 30 min), grey solid (CsA, 10 min), dark-blue solid (SCY-635, 60 min), blue dashed (SCY-635, 30 min), and light-blue solid (SCY-635, 10 min) lines represent the fitted lines. Closed (CsA) and open (SCY-635) circles (10 min), triangles (30 min), and squares (60 min) denote data mean ± SEM (*N* = 3 in triplicate) at different preincubation times following CsA or SCY-635 treatment. (**B**) *Trans*-inhibition assay condition used in (**A**). Cells were preincubated with vehicle control or an inhibitor, after washing cells, the uptake of [^3^H]-E_2_17βG was determined in the absence of the inhibitor.

Preincubation with CsA (0.1–10 µM) significantly reduced OATP1B1-mediated transport at all pretreatment times starting as early as 10 min (*p* < 0.00001 by mixed-effect model). Preincubation with SCY-635 significantly reduced OATP1B1-mediated transport at all preincubation times but only after preincubation at 5 and 10 µM (*p* < 0.00001 by mixed-effect model). As shown in Figure 2A and Table 1, preincubation with CsA (0.001–10 µM) yielded IC_50_ values of 0.30 ± 0.06, 0.16 ± 0.05, and 0.12 ± 0.04 µM (best fit values ± associated SE, *N* = 3 in triplicate) after preincubation for 10, 30, and 60 min, respectively. The IC_50_ values against OATP1B1 after SCY-635 (0.001–10 µM) preincubation for 10 and 30 min were approximately 9.4 and 3.1 µM, respectively. Preincubation with SCY-635 for 60 min yielded IC_50_ of 2.2 ± 1.4 µM against OATP1B1, which is ~18-fold greater than that by CsA.

### 3.2. Pretreatment Effects of Tacrolimus on OATP1B1- and OATP1B3-Mediated Transport

**Figure 3:**
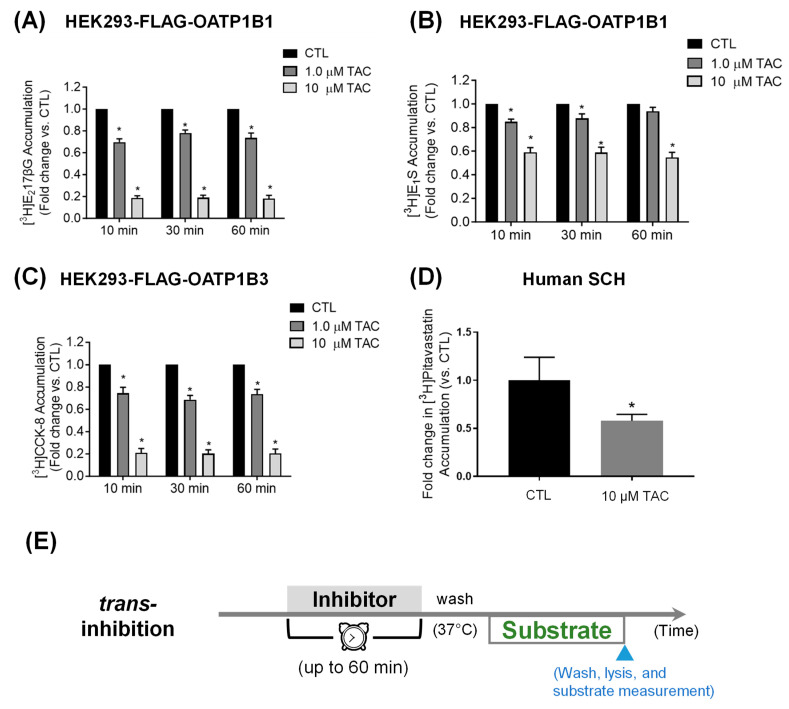
Effects of preincubation with tacrolimus on OATP1B1- and OATP1B3-mediated transport in transporter-expressing cell line and on pitavastatin accumulation in human SCH. Model-estimated fold change and associated SE of the accumulation of [^3^H]-E_2_17βG (1 µM, 2 min) (**A**), [^3^H]-E_1_S (1 µM, 2 min) (**B**), and [^3^H]-CCK-8 (1 µM, 3 min) (**C**) versus control (CTL) in HEK293-FLAG-OATP1B1 (**A**,**B**) and HEK293-FLAG-OATP1B3 (**C**) cells after preincubation with 1 and 10 µM tacrolimus (TAC) for 10, 30, or 60 min. Substrate accumulation was determined in the absence of tacrolimus after washing. Linear mixed effects models were fit to the data as described in Section 2 (*N* ≥ 3 in triplicate). * indicates a statistically significant difference (Bonferroni-adjusted *p* < 0.05) versus CTL. (**D**) [^3^H]-pitavastatin accumulation (1 µM, 0.5 min) in human SCH preincubated with tacrolimus (10 µM, 1 h) or vehicle control. After preincubation and washing, [^3^H]-pitavastatin accumulation was determined in the absence of the inhibitor. Data represents mean ± SD (*N* = 1 donor in triplicate). * indicates a statistically significant difference (*p* < 0.05) versus CTL by Student’s *t*-test. (**E**) *Trans*-inhibition assay condition used in (**A**–**D**).

Using E_2_17βG as the substrate, preincubation with tacrolimus (1 and 10 µM) for 10, 30, and 60 min significantly decreased OATP1B1-mediated [^3^H]-E_2_17βG transport (1 µM, 2 min) in all conditions (*p* < 0.001), ranging from 0.70 ± 0.03 to 0.78 ± 0.03- and 0.18 ± 0.03 to 0.19 ± 0.02-fold that of the control for 1 and 10 µM tacrolimus preincubation, respectively (Figure 3A). Using E_1_S as the substrate, preincubation with tacrolimus at 1 µM significantly (*p* < 0.001) decreased OATP1B1-mediated transport of [^3^H]-E_1_S (1 µM, 2 min) to 0.85 ± 0.02 and 0.88 ± 0.04-fold that of the control at 10 and 30 min, respectively; tacrolimus preincubation at 10 µM significantly decreased (*p* < 0.001) OATP1B1-mediated transport of [^3^H]-E_1_S (1 µM, 2 min) to 0.59 ± 0.04, 0.59 ± 0.05, and 0.55 ± 0.04-fold that of the control at 10, 30, and 60 min, respectively (Figure 3B). Under each condition, the preincubation-induced inhibitory effects of tacrolimus on OATP1B1-mediated transport was greater using E_2_17βG as the substrate than when using E_1_S. 

For OATP1B3, preincubation with tacrolimus (1 and 10 µM) for 10, 30, and 60 min significantly decreased OATP1B3-mediated [^3^H]-CCK-8 transport (1 µM, 3 min) under all conditions (*p* < 0.001), ranging from 0.69 ± 0.04 to 0.75 ± 0.05- and 0.20 ± 0.02 to −0.21 ± 0.04-fold that of the control for 1 and 10 µM tacrolimus preincubation, respectively (Figure 3C). 

The preincubation effects of tacrolimus on [^3^H]-pitavastatin accumulation was also observed in human SCH, wherein 1 h preincubation with tacrolimus (10 µM) significantly decreased [^3^H]pitavastatin accumulation to 0.58 ± 0.07-fold that of the control (*p* < 0.05 by Student’s *t*-test) (Figure 3D and Figure S1). 

### 3.3. Confocal Live Cell Imaging

**Figure 4:**
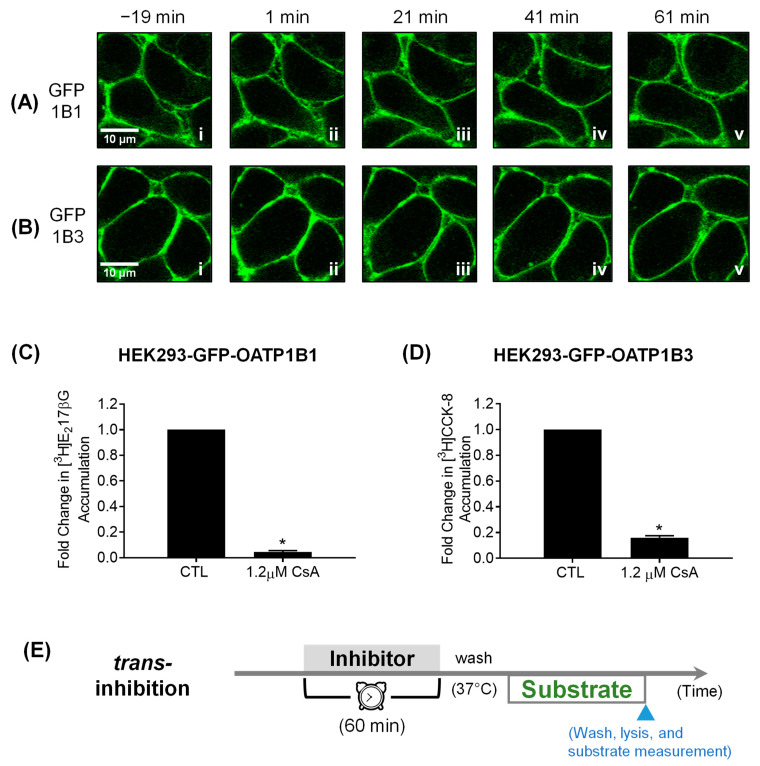
Time-lapse confocal microscopy and determining GFP-OATP1B1 and GFP-OATP1B3-mediated transport in HEK293-GFP-OATP1B1 and -GFP-OATP1B3 stable cell lines, respectively. (**A**) HEK293-GFP-OATP1B1 (GFP-1B1) and (**B**) GFP-OATP1B3 (GFP-1B3) stable cell lines were seeded at a density of 2.5 × 10^5^ cells per compartment in a 4-compartment culture dish and were grown for 48 h at 37 °C. At 48 h, dishes were transferred to and incubated in the Olympus Fluoview FV10i-LIV confocal laser-scanning microscope at 37 °C (95% O_2_, 5% CO_2_). The “zero” time point is the time of the addition of CsA to the cell culture medium to a final concentration of 1.2 µM. Images were taken every 20 min, from 19 min before the addition of CsA (−19 min) to 61 min after CsA treatment (61 min). Cells from three independent experiments were examined (*N* = 3). The pictures show representative z-stacks of three consecutive sections. For uptake experiments, HEK293-GFP-OATP1B1 (**C**) and -OATP1B3 cells (**D**) were seeded in 24-well plates at a density of 2.5 × 10^5^ cells per well and were allowed to grow to confluence for 48 h after seeding. (**C**) Model-estimated fold change and associated *SE* of [^3^H]E_2_17βG accumulation (1 µM, 2 min) versus CTL in HEK293-GFP-OATP1B1 after CsA treatment (1.2 μM, 1 h). (**D**) Model-estimated fold change and associated *SE* of [^3^H]-CCK-8 accumulation (1 µM, 3 min) versus CTL in HEK293-GFP-OATP1B3 after CsA treatment (1.2 μM, 1 h). Generalized linear mixed effects models were fit to the data (*N* = 3, in triplicate). * indicates a statistically significant difference (*p* < 0.01) versus CTL. (**E**) *Trans*-inhibition assay condition used in (**A**–**D**).

Utilizing HEK293-GFP-OATP1B1 and HEK293-GFP-OATP1B3 stably transfected cell lines, time-lapse confocal live cell imaging during CsA treatment revealed GFP-OATP1B1 and GFP-OATP1B3 after CsA treatment primarily localized to the plasma membrane with no discernable changes over the duration of treatment (Figure 4A,B). Preincubation with CsA (1.2 μM, 1 h) also significantly reduced OATP1B1- and OATP1B3-mediated transport of [^3^H]-E_2_17βG and [^3^H]-CCK-8 in GFP-OATP1B1- and GFP-OATP1B3-overexpressing stable cell lines (Figure 4C,D), respectively. 

### 3.4. Proteins associated with FLAG-OATP1B1 and FLAG-OATP1B3

**Figure 5:**
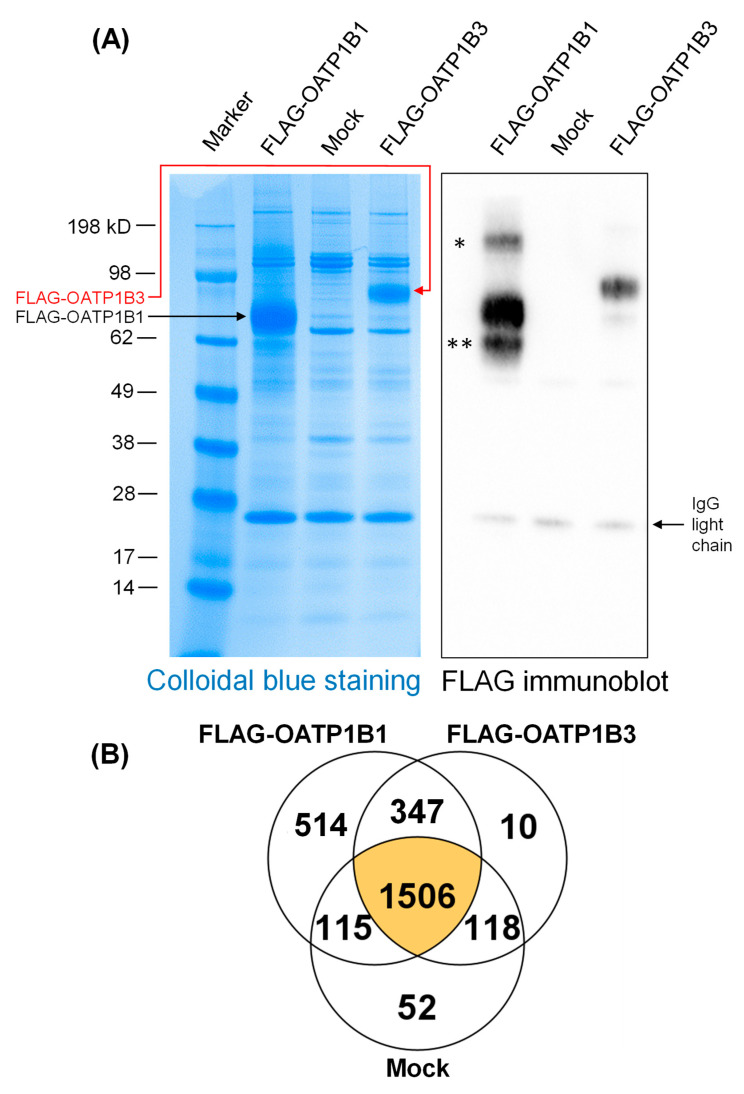
Immunoprecipitation of FLAG-OATP1B1 and FLAG-OATP1B3 and number of proteins identified in IP-MS. IP with FLAG antibody was conducted in whole cell lysates of HEK293-FLAG-OATP1B1, HEK293-FLAG-OATP1B3, and HEK293-Mock cells. Eluted immunocomplexes were resolved in SDS-PAGE (*N* = 1). (**A**) Colloidal blue staining (left panel) and FLAG immunoblot (right panel). Black and red arrows denote the major bands of purified OATP1B1 and OATP1B3, respectively. * and ** denote the higher and lower MW bands of OATP1B1 relative to the major band of OATP1B1. (**B**) Number of proteins associated with FLAG-IP in HEK293-FLAG-OATP1B1, FLAG-OATP1B3, and -Mock cells.

FLAG-IP has been widely used in IP-MS studies due to the existence of high-affinity FLAG antibodies, including the FLAG antibody used in the current study that is suitable for sensitive IP [71]. To validate samples used for IP-coupled LC-MS/MS for the global identification of proteins associated with FLAG-OATP1B1 and FLAG-OATP1B3, following FLAG-IP and subsequent washing and elution, an aliquot of the same eluted immunocomplexes used for proteomics was subjected to SDS-PAGE, Colloidal blue staining, and FLAG immunoblot. As shown in Figure 5A, left panel, FLAG-OATP1B1 (black arrow) and OATP1B3 (red arrow) were enriched from HEK293-FLAG-OATP1B1 and HEK293-FLAG-OATP1B3 cells, respectively, but not in the Mock cells. FLAG immunoblotting also verified the presence of FLAG-OATP1B1 and FLAG-OATP1B3 IP in HEK293-FLAG-OATP1B1 and HEK293-FLAG-OATP1B3 cells, respectively, but not in Mock cells (Figure 5A, right panel). In the current study, we lowered the temperature used to elute the immunocomplexes to 60 °C. This condition minimized the formation of the higher-molecular weight (MW) band of OATP1B1 (denoted on Figure 5A), which has been observed prominently in our previous studies, using boiling temperature to elute the immunocomplexes following IP [68]. A lower-MW band of OATP1B1 (denoted as ** in Figure 5A) was similar to that observed in our previous studies [12]. 

A total of 2482, 1982, and 1791 proteins were identified by FLAG-IP-MS/MS in HEK293-FLAG-OATP1B1, HEK293-FLAG-OATP1B3, and Mock cells, respectively. Among these, 861 and 357 proteins were specifically associated with FLAG-OATP1B1 and FLAG-OATP1B3, respectively, while they were not detected in Mock cells (Figure 5B). *Trans*-inhibition of OATP1B1 and/or OATP1B3 has been reported either previously or herein for the tyrosine kinase inhibitors (TKIs) nilotinib [13,14] and dasatinib [11], the mTOR kinase inhibitors sirolimus and everolimus [10,15], and the calcineurin and/or PPIase inhibitors CsA, tacrolimus, and SCY-635. Table 2 summarizes identified OATP1B1- and OATP1B3-accociated proteins related to biological processes and proteins relevant to these reported preincubation-induced inhibition effects. 

From the transporter regulation perspective, Table 2 summarizes identified OATP1B1- and OATP1B3-accociated proteins related to biological processes and proteins relevant to drugs with *trans*-inhibitory effects on OATP1B1/3 that are reported previously [8,16] or in the current study and are intuitively linked to a potential regulatory mechanism of OATP1B1/3. At present, the best-known factors involved in the regulation of OATP1B1/3 that are also applicable in the HEK293 system are the kinase activator [7,72] and inhibitor [13] compounds, as reviewed in the recent International Transporter Consortium white paper [66]. Hence, Table 2 currently focuses on kinase inhibitor drugs with reported *trans*-inhibitory effects on OATP1B1/3, as well as calcineurin and/or PPIase inhibitors from our current study. Table 3 and Table 4, respectively, summarize ubiquitination- related enzymes and protein kinases that were identified as associated with OATP1B1/3. Unless otherwise noted, all protein interactors described in the present work have been confirmed to be expressed at the protein level in human hepatocytes via resources publicly available at The Human Protein Atlas [73] and/or UniProt [74].

**Table 2:** OATP1B1- and OATP1B3-accociated proteins related to *trans*-inhibition of OATP1B1/3 by kinase inhibitor drugs and calcineurin/PPIase inhibitors.

*Types of Preincubation-Induced OATP1B1/1B3 Inhibition*		*OATP1B1- and OATP1B3-Interacting Proteins*					
Inhibitors (Affected Transporters, Reference)	Pharmacological Target(s) of Inhibitors, Reference	Relevant Identified Proteins	Accession Number(s)	Alternative Name(s)	OATP 1B1	OATP 1B3	Mock
**TKIs**	**Relevant Proteins**						
Nilotinib (OATP1B1 [13] and OATP1B3 [14])	Tyrosine Kinases **Lyn**, [13,14,75] **Fyn**, [75] **Yes**, [75] **MAP2k3** [75]	Tyrosine-protein kinases Lyn, Fyn, Yes, MAP2k3	LYN_HUMAN FYN_HUMAN YES_HUMAN MP2K3_HUMAN	LYN FYN YES1 MAP2K3	++ ++ ++ ++	++ ++ ++ -	- - - -
Dasatinib (OATP1B1 and OATP1B3) [11]	Tyrosine Kinases **Abl**, [75,76] **Bcr-Abl**, [76] **Lyn**, [75] **Fyn**, [75] **Yes**, [75] **Src** [75]	Abl interactor 2 Tyrosine-protein kinases Lyn, Fyn, Yes, Src	ABI2_HUMAN LYN_HUMAN FYN_HUMAN YES_HUMAN SRC_HUMAN	ABI2 LYN FYN YES1 SRC	+ ++ ++ ++ ++	- ++ ++ ++ ++	- - - - -
Pazopanib (OATP1B1) [65]	Tyrosine Kinases **Lyn**, [75] **Fyn**, [75] **Yes**, [75] **Src** [75]	Tyrosine-protein kinases Lyn, Fyn, Yes, Src	LYN_HUMAN FYN_HUMAN YES_HUMAN SRC_HUMAN	LYN FYN YES1 SRC	++ ++ ++ ++	++ ++ ++ ++	- - - -
**mTOR Inhibitors**	**Relevant Complexes**						
Sirolimus (OATP1B1 and OATP1B3) [10]	**mTORC1** [77]	Serine/threonine-protein kinase mTOR	MTOR_HUMAN	MTOR	++	+	-
Everolimus (OATP1B1 and OATP1B3) [10]	**mTORC1**, [77] **mTORC2** [78]	Serine/threonine-protein kinase mTOR	MTOR_HUMAN	MTOR	++	+	-
**calcineurin and/or PPIase Inhibitors**	**Relevant** **Immunophilins**						
CsA (OATP1B1 and OATP1B3) [4]	**Cyclophilin A** [79,80]	Peptidyl-prolyl cis-trans isomerase A (Cyclophilin A)	PPIA_HUMAN	PPIA	++	++	++
	**Cyclophilin B** [80]	Peptidyl-prolyl cis-trans isomerase B (Cyclophilin B)	PPIB_HUMAN	PPIB	++	++	++
Tacrolimus (OATP1B1 and OATP1B3, current study)	**FKBP5** [81]	Peptidyl-prolyl cis-trans isomerase FKBP5	FKBP5_HUMAN	FKBP5	++	-	-
	**FKBP8** [82]	Peptidyl-prolyl cis-trans isomerase FKBP8	FKBP8_HUMAN	FKBP8	++	+	-
SCY-635 (OATP1B1, current study)	**Cyclophilin A** [32]	Peptidyl-prolyl cis-trans isomerase A (Cyclophilin A)	PPIA_HUMAN	PPIA	++	++	++

“-“: total spectrum count of 0; “+”: total spectrum count (0–3); “++”: total spectrum count ≥ 3. Total spectrum counts are 0 in Mock for all entries in Table 2.

## 4. Discussion

Understanding the mechanisms underlying the regulation of OATP1B1 and OATP1B3 has clinical significance in mitigating OATP-mediated DDIs in drug therapy and in the rational design of drug candidates. The current study for the first time reported *trans*-inhibition of OATP1B1/3 by the CNI tacrolimus and a non-immunosuppressive cyclophilin inhibitor. Also, various proteins associated with OATP1B1 and OATP1B3 have been identified, of which many are related to known OATP1B1/3 regulatory mechanisms and/or preincubation-induced *trans*-inhibition by various drugs. 

Both CsA and its structurally similar analogue SCY-635 elicit preincubation-induced *trans*-inhibitory effects on OATP1B1 (Figure 2). During the 60 min preincubation period, both CsA and SCY-635 exhibited progressively increased *trans*-inhibitory effects on OATP1B1 with preincubation IC_50_ values decreased over time (Table 1). The *trans*-inhibition IC_50_ value of SCY-635 at 10 min could not be accurately estimated due to the less potent inhibition observed. Compared to respective IC_50_ values at 60 min preincubation, CsA has 2.5- and 1.3-fold higher preincubation IC_50_ values at 10 and 30 min, respectively; SCY-635 has 1.4-fold higher IC_50_ at 30 min. A similar trend of progressively reduced *trans*-inhibition IC_50_ of CsA has been reported over 5–120 min preincubation times [9]. To compare the *trans*-inhibitory effects of CsA and SCY-635 on OATP1B1 at each preincubation time, the same data in Figure 2 were replotted in Figure S2. At each preincubation time, CsA (0.1–10 µM) has greater inhibitory effects on OATP1B1 than SCY-635. 

We reevaluated the tacrolimus preincubation effects on OATP1B1- and OATP1B3-mediated transport, using a more sensitive substrate for OATP1B1, E_2_17βG, and demonstrated the *trans*-inhibition of both OATP1B1 and OATP1B3 by tacrolimus (Figure 3). The *trans*-inhibition of OATP1B1/3 rapidly reaches maximum inhibitory effect and does not change over the 60 min preincubation period at each tacrolimus concentration determined (Figure 3A–C). A similar type of *trans*-inhibition that lacks the progressive reduction of OATP1B1/3-mediated transport over the preincubation period has been reported for rifampicin [9,11]. The discrepancy in *trans*-inhibition of OATP1B1 by tacrolimus between the current study and the previous report [4] is likely due to the use of the less sensitive substrate E_1_S for OATP1B1 [60] and the apparent large variability observed in the previous study. *Trans*-inhibition by tacrolimus was also observed in physiologically relevant human SCH using the previously validated [83] OATP substrate pitavastatin as the probe substrate (Figure 3D). 

Although both CNIs, CsA and tacrolimus, exhibit *trans*-inhibition capability toward OATP1B1, our data suggest that calcineurin inhibition is not likely a prerequisite for their *trans*-inhibitory effects on OATP1B1. This is evident as the inactive analogue of CsA, SCY-635, lacking calcineurin inhibition activity, still elicits *trans*-inhibition of OATP1B1. However, not excluding other possible mechanism(s), calcineurin inhibition may contribute to the greater *trans*-inhibition potency of CsA compared to SCY-635 observed at each preincubation time of 10, 30, and 60 min (Figure S2). The function of phosphoproteins including NFAT and tau has been reported to be regulated by CsA via altered phosphorylation upon calcineurin inhibition [84]. As OATP1B1 [10,12] and OATP1B3 [7] are phosphorylated proteins, it is possible that the phosphorylation status and function of OATP1B1 [10,12] and OATP1B3 [7] are altered by CsA treatment via calcineurin inhibition. Utilizing a quantitative phosphoproteomics approach to identify the phosphorylation sites of OATP1B1 and OATP1B3 that are responsive to CsA and SCY-635 treatment could shed light on how calcineurin may be involved in modifying transport function, thereby warranting further investigation. Additionally, employing a calcineurin knockout or knockdown cell system will enable the specific evaluation of the role of calcineurin in CsA’s *trans*-inhibitory effects on OATP1B1, which also warrants further investigation.

Various mechanisms have been implicated to be involved in the *trans*-inhibition of OATP1B1/3. Our laboratory previously reported that the *trans*-inhibition of phorbol 12-myristate 13-acetate (PMA) on OATP1B3-mediated transport is associated with protein kinase C activation and the increased phosphorylation of OATP1B3 [7]. Many drugs included in Table 2 are kinase inhibitor drugs. This is consistent with a recent review in the International Transporter Consortium white paper [66] showing that kinase modulators are the most known post-translational mechanism involved in OATP1B1/3 regulation studied in the HEK293 system. Recent publications have shown that in addition to OATP1B1 and OATP1B3, *trans*-inhibition and/or time-dependent inhibition are observed for various solute carrier (SLC) uptake transporters such as OATs, OCTs, and multidrug and toxic compound extrusion (MATEs) [16,85,86,87,88], implying potential involvement of a common mechanism. The mechanisms underlying the common *trans*-inhibition and time-dependent inhibition of SLC transporters warrant investigation. 

Interestingly, several PPIases including FKBPs and cyclophilins were identified in association with OATP1B1 and/or OATP1B3, though only FKBP5 and FKBP8 were associated specifically with OATP1B1 and/or OATP1B3 apart from our Mock negative control (Table 2). It is noteworthy that the mTOR inhibitor sirolimus is also a PPIase inhibitor [89]. Previously reported *trans*-inhibition of OATP1B1/3 by sirolimus occurs via a currently unknown mechanism independent of its mTOR inhibition activity [10]. It is tempting to connect the shared PPIase inhibition activity of CsA, tacrolimus, and sirolimus with their *trans*-inhibition characteristics toward OATP1B1/3, although such a possibility requires further investigation. 

For CsA, long-lasting inhibitory effects have been reported in stably transfected OATP1B1-expressing HEK293T cells, wherein after CsA preincubation and washing, the OATP1B1-mediated transport remained reduced until at least 18 h [4]. We observed similar long-lasting inhibitory effects on pitavastatin uptake by tacrolimus in human SCH. After preincubation with CsA or tacrolimus (each at 10 µM) for 1 h and washing and re-culture in inhibitor-free medium for 2 h, pitavastatin accumulation was maintained at a significantly decreased level, measuring 0.49 ± 0.1- and 0.59 ± 0.09-fold that of the control for CsA and tacrolimus, respectively (Figure S1). Currently, tacrolimus is the primary immunosuppressant used by transplant recipients [53,54]. A case of severe rosuvastatin-related rhabdomyolysis has been reported approximately 1 month after switching to an intensified tacrolimus dosing regimen [59]. The inhibition of tacrolimus on rosuvastatin uptake in human SCH, including its *trans*-inhibition and long-lasting inhibition characteristics, is worth further investigation to elucidate the clinically relevant potential of OATP-mediated DDIs via tacrolimus. Additionally, several non-immunosuppressive CsA analogues lacking calcineurin inhibition activity have also been tested for clinical implications [32,49,50,51]. We would anticipate such non-immunosuppressive cyclophilin inhibitors likely have relatively mild preincubation-induced inhibitory effects on OATP1B1 as observed for SCY-635. Future correlation of such preincubation-induced inhibitory effects with clinical DDI studies between CsA and non-immunosuppressive PPIase inhibitors may shed more light on the role of calcineurin inhibition in clinical OATP-mediated DDIs. 

Our IP-MS proteomics data identified several OATP1B1- and OATP1B3-associated proteins consistent with a diverse assortment of previous regulatory studies. Lyn kinase has been previously described as a regulator of both OATP1B1 [13] and OATP1B3 [14] transport functions. The inhibition of Lyn kinase activity by preincubation with nilotinib or knockdown of Lyn reduces OATP1B1- and OATP1B3-mediated transport [13,14], respectively, and is associated with a concomitant reduction in the phosphorylation of OATP1B1 at several tyrosine residues [13]. In agreement with these findings, the present proteomics data detected specific interaction of Lyn with both OATP1B1 and OATP1B3 (Table 2 and Table 4). Dasatinib potently inhibits the constitutively active fusion gene product Bcr-Abl kinase [90], and preincubation with the drug significantly reduces OATP1B1- and OATP1B3-mediated transport under *trans*-inhibition conditions [11,15]. Relevant to these previous findings, we detected an association of Abl Interactor 2 (ABI2), a regulator of Abl kinase [76], with OATP1B1. In addition, kinases inhibited by pazopanib were also identified for the first time in our proteomics data (Table 2) [75].

mTOR is a regulatory kinase with diverse functions [91,92]. Both sirolimus and everolimus can *trans*-inhibit OATP1B1/1B3 independent of their mTOR kinase inhibition activity [10]. Relevantly, mTOR and several related regulatory proteins were identified to be associated with OATP1B1/3 (Table 2 and Table S2). These include mTOR, mTORC1 subunit RPTOR [93,94], mTORC1 and mTORC2 assembly factor TTI1 [95], and LAMTOR1, a scaffold protein required to activate mTORC1 [96]. The specific implications of these interactions are unknown, but these findings combined with the previous observations [10] could hint at an interaction-based regulatory mechanism for OATP1B1/1B3 by mTOR that is independent of mTOR’s kinase activity.

Ubiquitination is catalyzed in tandem by the ubiquitin (Ub)-activating enzyme (E1), ubiquitin-conjugating enzyme (E2), and ubiquitin ligase (E3), whereas deubiquitination is mediated by DUBs. Enzymes involved in the ubiquitination and deubiquitination of OATP1B1 and OATP1B3 have not been previously reported. The current study identified 2 E2s, 13 E3s, and 2 DUBs associated with OATP1B1 and/or OATP1B3 and not detected in Mock (Table 3). Furthermore, we identified 28 protein kinases (Table 4), including several Src family tyrosine kinases, in association with OATP1B1 and/or OATP1B3. These findings represent potential regulatory processes governing the transport function of OATP1B1 and OATP1B3. 

While calcineurin itself was not detected in our IP-MS dataset, calcineurin is known to dephosphorylate or otherwise interact with several proteins that were identified associated with OATP1B1 and OATP1B3. Previously confirmed calcineurin substrates or binding partners of note within our IP-MS dataset are as follows: CDC20 [97], Caveolin-1 [98], and RACK1 [99,100] (Table S2). These interacting proteins represent potential candidate interactors by which changes to calcineurin activity could indirectly alter OATP1B1/3 transport activity via changes to these interactors’ own activities, though decisive conclusions about this matter are beyond the scope of this work. 

In addition to OATP1B1 and OATP1B3, *trans*-inhibition and/or time-dependent inhibition have been reported for various solute carrier uptake transporters such as OATs, OCTs, and multidrug and toxic compound extrusion (MATEs) [16,85,86,87,88], likely implying the involvement of one or more common mechanisms. Such a common mechanism does not exclude the possibility of shared, yet unidentified biological processes contributing to these similarities. Our study does not undermine the importance of other previously reported factors, such as the slow build-up of unbound intracellular inhibitor concentrations, as potential mechanisms underlying the commonality among diverse time-dependent inhibitors [9,16].

The current studies identified proteins interacting with OATP1B1 and OATP1B3 in a constitutive condition. Such interactions provide novel insights into enzymes and biological processes that may regulate the transport function of OATP1B1 and OATP1B3. Individual interactions of special interest remain to be further validated by another method such as IP followed by immunoblotting or by additional IP-MS biological replicates. Thus, individual IP-MS interactions should currently be interpreted with due caution. Our findings, for the first time, identified that several proteins related to preincubation-induced *trans*-inhibition of OATP1B1 and OATP1B3 likely directly interact with the transporters under basal conditions without drug treatments. The HEK293 stable cell lines used in the current studies are of kidney origin and thus are not perfectly representative of endogenous OATP1B1 and OATP1B3 expression in hepatocytes in vivo. Interestingly, however, the majority of the identified FLAG-OATP1B1 and FLAG-OATP1B3-associated proteins (unless otherwise specified in Table 3 and Table 4) have known expression in human liver, as validated via the publicly available databases The Human Protein Atlas [73] and/or UniProt [74]. It would be of further interest to use a similar quantitative proteomics approach to compare changes of association in the presence of various drugs with known OATP1B1/3 *trans*-inhibitory effects and in a more physiologically relevant cell type such as primary human hepatocytes. 

## Figures and Tables

**Table 1:** Preincubation IC_50_ values against OATP1B1 elicited by CsA and SCY-635.

Preincubation Time	IC_50_ (µM)	
	CsA	SCY-365
10 min	0.30 ± 0.06	9.4 ± 16.3
30 min	0.16 ± 0.05	3.1 ± 3.3
60 min	0.12 ± 0.04	2.2 ± 1.4

HEK293-FLAG-OATP1B1 cells were preincubated with CsA or SCY-635 each at 0.001–10 µM in DMEM complete medium containing 10% FBS and 1% antibiotic antimycotic for 10, 30, or 60 min. After washing with HBSS buffer, OATP1B1-mediated [^3^H]E_2_17βG was determined. IC_50_ values are expressed as the mean ± SE from model estimation (*N* = 3 in triplicate).

**Table 3:** Ubiquitination enzymes and deubiquitinases (DUBs) associated with OATP1B1 and OATP1B3.

Identified Proteins	Accession Number	Alternative Name	OATP 1B1	OATP 1B3
**Ubiquitin-conjugating enzymes (E2)**				
Ubiquitin-conjugating enzyme E2 Q1 ^a^	UB2Q1_HUMAN	UBE2Q1	++	-
Ubiquitin-conjugating enzyme E2 G2	UB2G2_HUMAN	UBE2G2	++	-
**Ubiquitin ligases (E3)**				
E3 ubiquitin-protein ligase RNF5	RNF5_HUMAN	RNF5	++	+
E3 ubiquitin-protein ligase HECTD1	HECD1_HUMAN	HECTD1	++	-
E3 ubiquitin-protein ligase AMFR	AMFR_HUMAN	AMFR	++	-
E3 ubiquitin-protein ligase Itchy homolog	ITCH_HUMAN	ITCH	++	-
E3 ubiquitin-protein ligase listerin	LTN1_HUMAN	LTN1	++	-
E3 ubiquitin-protein ligase RNF126 ^a^	RN126_HUMAN	RNF126	++	+
E3 ubiquitin-protein ligase TRIM32	TRI32_HUMAN	TRIM32	++	-
Ubiquitin-protein ligase E3C	UBE3C_HUMAN	UBE3C	++	-
E3 ubiquitin-protein ligase Praja-1 ^a^	PJA1_HUMAN	PJA1	++	+
Ubiquitin conjugation factor E4 A	UBE4A_HUMAN	UBE4A	++	-
Mitochondrial ubiquitin ligase activator of NFKB 1	MUL1_HUMAN	MUL1	+	-
Probable E3 ubiquitin-protein ligase HERC1	HERC1_HUMAN	HERC1	+	-
E3 ubiquitin-protein ligase UBR3	UBR3_HUMAN	UBR3	++	-
**Deubiquitinases (DUBs)**				
Ubiquitin carboxyl-terminal hydrolase 24	UBP24_HUMAN	USP24	+	+
Ubiquitin carboxyl-terminal hydrolase isozymeL1 ^a^	UCHL1_HUMAN	UCHL1	++	+
Ubiquitin thioesterase OTUB1	OTUB1_HUMAN	OTUB1	++	-

“-“: total spectrum count of 0; “+”: total spectrum count (0–3); “++”: total spectrum count ≥ 3. Total spectrum counts are 0 in Mock for all entries in Table 3. **^a^** denotes proteins without known expression in human hepatocytes.

**Table 4:** Protein kinases associated with OATP1B1 and OATP1B3.

Identified Proteins	Accession Number	Alternative Name	OATP 1B1	OATP 1B3
Tyrosine-protein kinase Yes	YES_HUMAN	YES1	++	++
Tyrosine-protein kinase Fyn	FYN_HUMAN	FYN	++	++
Tyrosine-protein kinase Lyn	LYN_HUMAN	LYN	++	++
Cyclin-dependent kinase 2	CDK2_HUMAN	CDK2	++	-
Cyclin-dependent kinase 4	CDK4_HUMAN	CDK4	++	-
Proto-oncogene tyrosine-protein kinase Src	SRC_HUMAN	SRC	++	++
Non-receptor tyrosine-protein kinase TYK2	TYK2_HUMAN	TYK2	++	-
Serine/threonine-protein kinase VRK2	VRK2_HUMAN	VRK2	++	-
Serine/threonine-protein kinase mTOR	MTOR_HUMAN	MTOR	++	+
Serine/threonine-protein kinase Nek7	NEK7_HUMAN	NEK7	++	+
Casein kinase I isoform gamma-3	KC1G3_HUMAN	CSNK1G3	++	++
Dual specificity mitogen-activated protein kinase kinase 3	MP2K3_HUMAN	MAP2K3	++	-
Serine/threonine-protein kinase LMTK2	LMTK2_HUMAN	LMTK2	++	-
Serine/threonine-protein kinase 17A	ST17A_HUMAN	STK17A	++	-
Cyclin-G-associated kinase	GAK_HUMAN	GAK	++	+
STE20/SPS1-related proline-alanine-rich protein kinase	STK39_HUMAN	STK39	++	-
G protein-coupled receptor kinase 6	GRK6_HUMAN	GRK6	+	+
RAC-beta serine/threonine-protein kinase	AKT2_HUMAN	AKT2	++	+
Serine/threonine-protein kinase TNNI3K ^a^	TNI3K_HUMAN	TNNI3K	++	-
3-phosphoinositide-dependent protein kinase 1	PDPK1_HUMAN	PDPK1	++	++
Phosphatidylinositol 4,5-bisphosphate 3-kinase catalytic subunit alpha isoform	PK3CA_HUMAN	PIK3CA	+	+
Serine-protein kinase ATM	ATM_HUMAN	ATM	++	-
Receptor tyrosine-protein kinase erbB-2	ERBB2_HUMAN	ERBB2	++	-
Serine/threonine-protein kinase SMG1	SMG1_HUMAN	SMG1	++	-
Serine/threonine-protein kinase 19 ^a^	STK19_HUMAN	STK19	+	-
TGF-beta receptor type-1	TGFR1_HUMAN	TGFBR1	++	++
Bone morphogenetic protein receptor type-2	BMPR2_HUMAN	BMPR2	++	+
Cell division cycle 7-related protein kinase	CDC7_HUMAN	CDC7	+	-

“-“: total spectrum count of 0; “+”: total spectrum count (0–3); “++”: total spectrum count ≥ 3. Total spectrum counts are 0 in Mock for all entries in Table 4. **^a^** denotes proteins without known expression in human hepatocytes.

## Data Availability

Data are contained within the article or Supplementary Materials.

## Supplementary Materials

The following supporting information can be downloaded at: https://www.mdpi.com/article/10.3390/pharmaceutics16010063/s1, Figure S1: Long-lasting pitavastatin uptake in human SCH following tacrolimus and CsA treatment; Table S1: Demographics of human hepatocytes donors; Table S2: Calcineurin substrates associated with OATP1B1 and OATP1B3; Table S3. Proteomics datastet.
