# Supplementary material for: Assessing Trans-Inhibition of OATP1B1 and OATP1B3 by Calcineurin and/or PPIase Inhibitors and Global Identification of OATP1B1/3-Associated Proteins
Source: Pharmaceutics. 2023 Dec 31;16(1):63. doi: 10.3390/pharmaceutics16010063 (PMC10818623; doi:10.3390/pharmaceutics16010063)
Supplement: Supplementary file 1 [file pharmaceutics-16-00063-s001.zip › pharmaceutics-2629617-supplementary.pdf]

**Supplemental Files**

## Assessing Trans-Inhibition of OATP1B1 and OATP1B3 by Calcineurin and/or PPIase Inhibitors and Global Identification of OATP1B1/3-Associated Proteins

John T. Powell, Ruhul Kayesh, Alexandra Ballesteros-Perez, Khondoker Alam, Pascaline Niyonshuti, Erik J. Soderblom, Kai Ding, Chao Xu, Wei Yue

Table S1. **Demographics of human hepatocytes donors**

| Donors | Age (years) | Gender | Race | BMI | Smoking | Alcohol use |
|---|---|---|---|---|---|---|
| HUM181621 | 23 | F | Caucasian | 31.3 | No | once per year |

F, female; M, male; BMI, body mass index

A

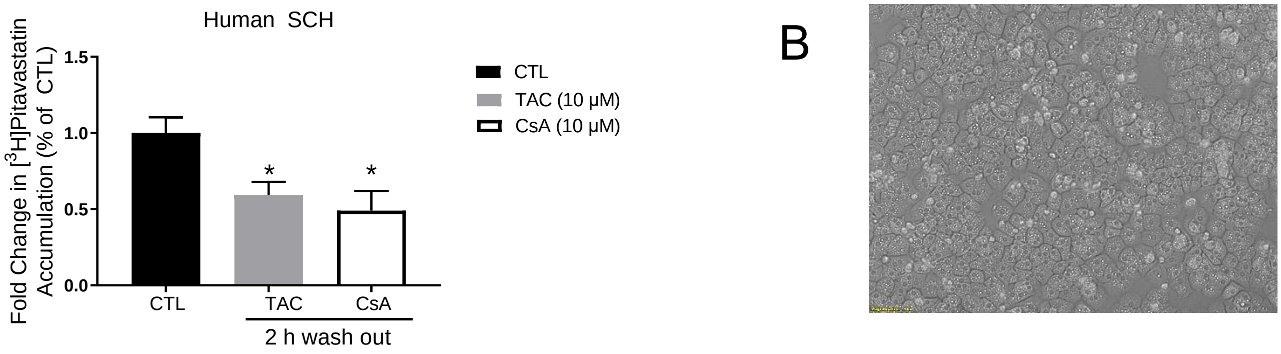


**Figure S1** Long-lasting pitavastatin uptake in human SCH following tacrolimus and CsA treatment. Human SCH were pretreated with CsA or tacrolimus (each 10 μM for 1 h). After washing, cells were cultured in inhibitor-free culture medium, [$^3$H]-pitavastatin accumulation (1 μM, 30 sec) was determined at 3 h after wash out phase in the drug-free medium. Data represent mean ± SD in triplicate. * $p<0.05$ by one way ANOVA vs. CTL. (B) Representative light images of human SCH on the day of experiment (10X).

**Table S2. Calcineurin- and mTOR-relevant proteins associated with OATP1B1 and OATP1B3.**

| Identified Proteins | Accession Number | Alternative Name | OATP 1B1 | OATP 1B3 |
|---|---|---|---|---|
| **Calcineurin substrates** | | | | |
| Cell division cycle protein 20 homolog | CDC20_HUMAN | CDC20 | + | + |
| Caveolin-1 | CAV1_HUMAN | CAV1 | ++ | ++ |
| **mTOR-relevant proteins** | | | | |
| Receptor of activated protein C kinase 1 | RACK1_HUMAN | RACK1 | ++ | ++ |
| Regulatory-associated protein of mTOR | RPTOR_HUMAN | RPTOR | ++ | - |
| TELO2-interacting protein 1 | TTI1_HUMAN | TTI1 | ++ | + |
| Ragulator complex protein LAMTOR1 | LTOR1_HUMAN | LAMTOR1 | ++ | ++ |

"-": total spectrum count of 0; "+": total spectrum count (0-3); "++": total spectrum count ≥3
Total spectrum counts are 0 in Mock for all entries in Table S1.
